# Supplementary material for: An Integrative miRNA-mRNA Expression Analysis Reveals Striking Transcriptomic Similarities between Severe Equine Asthma and Specific Asthma Endotypes in Humans
Source: Genes (Basel). 2020 Sep 28;11(10):1143. doi: 10.3390/genes11101143 (PMC7600650; doi:10.3390/genes11101143)
Supplement: Supplementary file 1 [file genes-11-01143-s001.zip › Supplementary Figures S1-S4.docx]

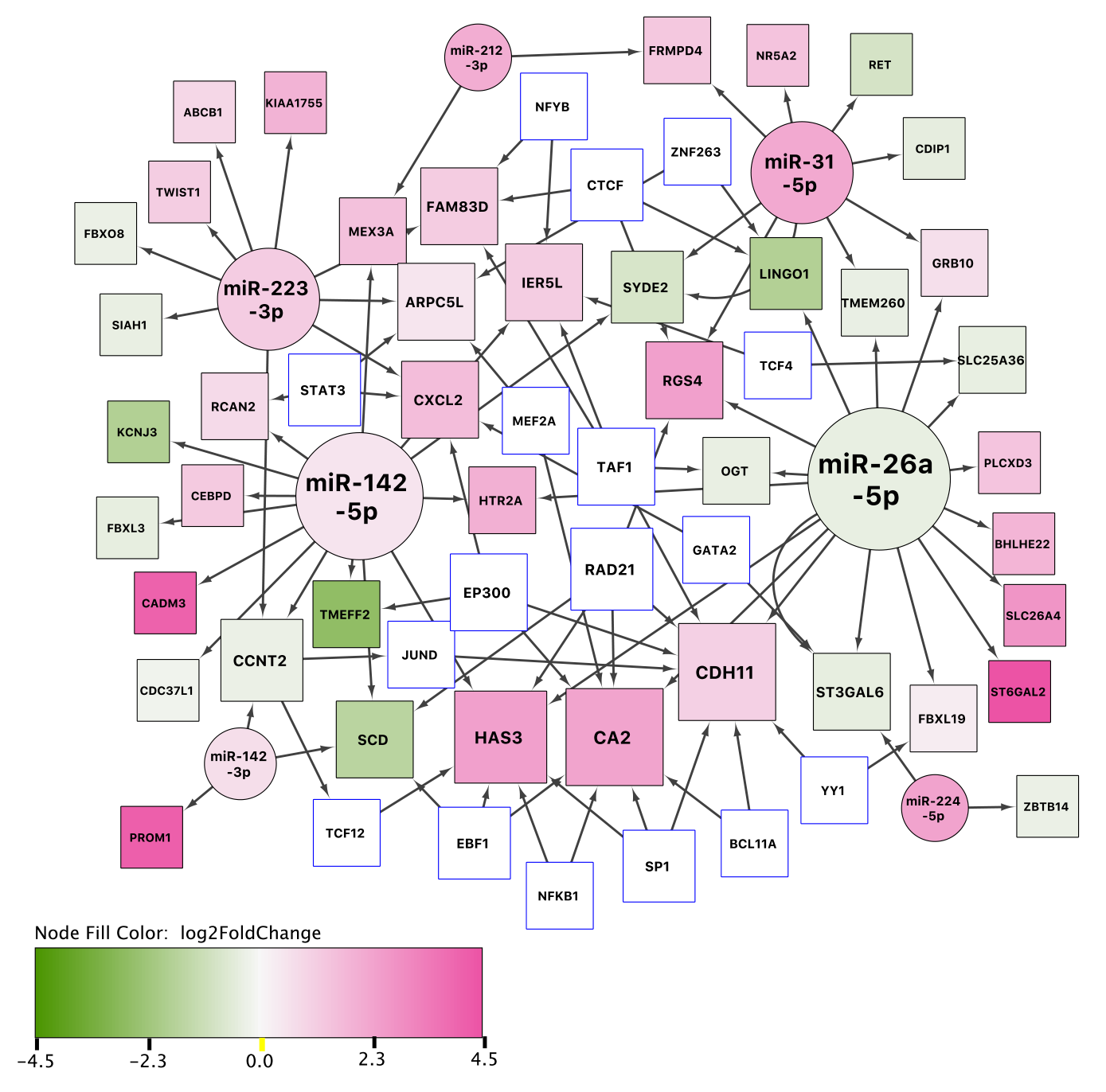


**Supplementary Figure S1: miRNA-gene interaction network exacerbation vs. controls.** The significant miRNAs and genes of the first contrast (exacerbation vs. controls) were used. In this version of the network, we expanded the initial network of just differentially expressed genes and miRNAs with transcription factors (represented as squares with white filling and blue border), to potentially increase the connectivity of the resulting network.

**
Supplementary Figure S3: miRNA-gene interaction network with transcription factors (exacerbation vs. remission).** The significant miRNAs and genes of the third contrast (exacerbation vs. remission) were used. In this version of the network, we expanded the initial network of just differentially expressed genes and miRNAs with transcription factors (represented as squares with white filling and blue border), to potentially increase the connectivity of the resulting network**.**

**Supplementary Figure S2: miRNA-gene interaction network exacerbation vs. controls with pathways.** The significant miRNAs and genes of the first contrast (exacerbation vs. controls) were used. In this version of the network, we expanded it not only with transcription factors but also pathways, to potentially assist with biological interpretation of the results.

**
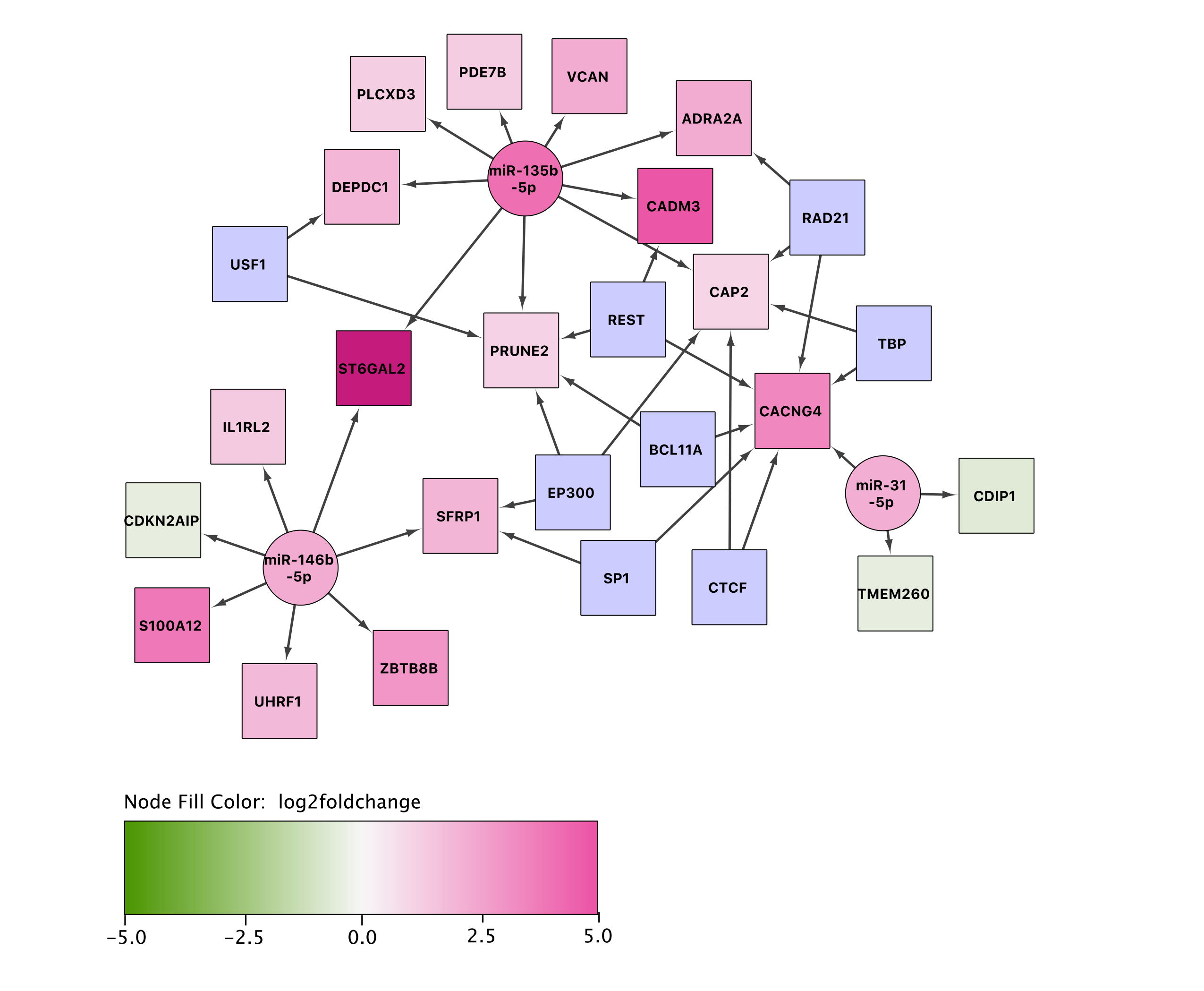
**

**Supplementary Figure S4: miRNA-gene interaction network with pathways (exacerbation vs. remission).** The significant miRNAs and genes of the third contrast (exacerbation vs. remission) were used. In this version of the network, we expanded the network with pathways, to potentially assist with biological interpretation of the results.
