# Supplementary material for: An Integrative miRNA-mRNA Expression Analysis Reveals Striking Transcriptomic Similarities between Severe Equine Asthma and Specific Asthma Endotypes in Humans
Source: Genes (Basel). 2020 Sep 28;11(10):1143. doi: 10.3390/genes11101143 (PMC7600650; doi:10.3390/genes11101143)
Supplement: Supplementary file 1 [file genes-11-01143-s001.zip › Supplementary Notes S1-S5.docx]

**Supplementary Note S1: Study population.**

|  | Exacerbation (n = 6) | Remission (n = 5) | Controls (n =8) |
| --- | --- | --- | --- |
| Age (mean ± sd) | 18.3 y ± 5.2 y | 21 y ± 4.8 y | 17.5 y ± 2 y |
| Sex | 3 mares / 3 geldings | 4 mares / 1 gelding | 6 mares / 2 geldings |
| Breeds | Appaloosa (1)  Quarter Horse (2)  Canadian Horse (1)  Arabian (1)  Cross (1) | Standardbred (1)  Quarter Horse (1)  Percheron (1)  Cross (2) | Standardbred (4)  Canadian (1)  Cross (1)  Quarter Horse (1)  Arabian (1) |

**Supplementary Note S2: Sequencing quality control.**

High-throughput small RNA sequencing yielded a mean of 12.2 million reads (± 2 million reads standard deviation). Initial quality control of the reads using FastQC showing a mean Phred score of Q35 throughout all reads, indicating that the sequences obtained are of high quality.

Analyzing the read length distribution pattern of all reads obtained, confirmed the successful enrichment of miRNAs, as the clear peak appeared around 22 nucleotides (nts) and was ranging from 19-24 nts, corresponding to the average size distribution of miRNAs[1].

Mapping the quality and adapter trimmed reads to the current equine reference genome EquCab3.0 using the mapper.pl script by miRDeep2 showed a mean mapping percentage of 71,5% (± 7.5% standard deviation).

Applying our previously established and published state-of-the-art bioinformatics pipeline for detecting high-confidence novel miRNAs[2], we were able to detect 283 putative high-confidence novel equine miRNAs. To achieve this a stringent miRDeep2 score cutoff of >= 5 was applied (as both the signal-to-noise ratio as well as the percentage of estimated true positives started to drop considerably below a miRDeep2 score of 5). Additionally, one putative novel miRNA was excluded due to a Rfam (rRNA/tRNA) hit, suggesting it to be a false positive miRNA.

**Supplementary note S3: Pathway analysis of differentially expressed miRNAs and their target genes.**

The KEGG pathway “TGF-beta signaling” was significantly (P_adjusted_ = 1.72*10^-7^) enriched with 35 target genes and three miRNAs being a part of it (*TGFBR1, RHOA, SMAD2, SMAD4, SMAD5, SMAD1 MYC, BMP8A, TGFB3, etc.*). Additionally, the pathway “PI3K-Akt signaling” was significantly (P_adjusted_ = 0.02) enriched with 96 target genes being a part of it together with all seven differentially expressed miRNAs. Some additional interesting significantly enriched pathways were “FoxO signaling” (P_adjusted_ P = 0.0003; 54 target genes), “TNF signaling” (P_adjusted_ = 0.0003, 41 target genes), “Bacterial invasion of epithelial cells” (P_adjusted_ = 0.0016, 30 target genes), and “T cell receptor signaling” (P_adjusted_ = 0.042, 36 target genes), while the pathway presenting the lowest p-value was “Hippo signaling” (P_adjusted_ = 4.95*10^-11^, 56 target genes) (Supplementary Table S2).

Pathway analysis using the six known significantly differentially expressed miRNAs between asthmatic horses in remission and control horses yielded following potentially interesting enriched pathways: “mTOR signaling” (P_adjusted_ = 0.00013, 22 target genes), “TNF signaling” (P_adjusted_ = 0.024, 27 target genes), “FoxO signaling” (P_adjusted_ = 0.031, 31 target genes), “Bacterial invasion of epithelial cells” (P_adjusted_ = 0.038, 18 target genes), “PI3K-Akt signaling” (P_adjusted_ = 0.04, 58 target genes), and “B cell receptor signaling” (P_adjusted_ = 0.04, 18 target genes) (Supplementary Table S3).

In the pathway analysis for the last differential miRNA expression analysis between asthmatic horses in an exacerbated state and asthmatic horses in remission (using the three known significant miRNAs), some potentially relevant and significantly enrich pathways were: “Lysine degradation” (P_adjusted_ = 0.0006, 8 target genes), “Hippo signaling” (P_adjusted_ = 0.0023, 24 target genes), “Bacterial invasion of epithelial cells” (P_adjusted_ = 0.011, 12 target genes), “Shigellosis” (P_adjusted_ = 0.0029, 14 target genes), and “TNF signaling” (P_adjusted_ = 0.0029, 17 target genes) (Supplementary Table S4).

**Supplementary Note S4: mRNA sequencing data processing.**

Quantification as well as quality control of the extracted mRNA fraction was confirmed using a Bioanalyzer system as well as a Qubit device. Even though there is no universal agreement about an RNA integrity number (RIN) cutoff value, it is generally accepted to employ a RIN cutoff value of 6, while a RIN of above eight is generally considered to be optimal[3,4]. Our extracted large RNA fractions showed a mean RIN of 8.13 (± 0.6 sd), the lowest value obtained was a RIN of 6.4 for one sample, while only four samples in total showed a value under 7.9. A first analysis of the resulting sequencing reads showed that we were able to obtain a mean of 38.8 million reads per sequencing library (± 1.8 million reads SD). Analyzing the base call quality scores showed that all reads showed a Phred score of at least Q31, while the average quality score lied around Q39. After removing remaining adapter sequences as well as performing quality trimming of the sequencing reads, we aligned the reads to the equine reference genome EquCab3.0 using the read aligning tool STAR.

Applying this approach, we achieved a mean percentage of uniquely mapped reads of 90.4% (±1.1% SD) meaning that a mean absolute amount of 35.2 million reads (± 2 million reads SD) per library was successfully mapped to the equine genome.

After quantifying the mRNA expression levels with the tool htseq-count[5], we conducted differential expression analyses using the R package DESeq2. As a first prefiltering step to quantitatively assess gene expression we required every gene to show non-zero counts in at least 90% of the samples in order to be considered expressed. We detected 20’288 genes to be considered expressed according to our definition in the 19 samples.

**Supplementary Note S5: Differentially expressed mRNA implicated in human asthma.**

Intersecting this set of differentially expressed genes with a set of genes that have been previously associated with human asthma from the database disGeNET v6.0, a large and expert-curated database of genes and variants associated with human diseases, revealed that of the significantly differentially expressed genes (exacerbation vs. healthy) 46 have been previously associated with asthma and might therefore be promising candidate genes for further research. The top 12 asthma associated genes according to the disGeNET “gene-disease association score”, which considers the number of studies, the level of curation and the number of organisms studied, were: *NPY, CXCL14, IL4R, MS4A2, ADAMTS14, SLC7A2, VDR, NOS3, SLC26A4, CXCL10, FCER1A, ITGB3* (Supplementary Table S6).

**Bibliography:**

1. DP, B. MicroRNAs: genomics, biogenesis, mechanism, and function. *Cell* **2004**, *116*, 281–297.

2. Pacholewska, A.; Kraft, M. F.; Gerber, V.; Jagannathan, V. Differential expression of serum MicroRNAs supports CD4+t cell differentiation into Th2/Th17 cells in severe equine asthma. *Genes (Basel).* **2017**, *8*, doi:10.3390/genes8120383.

3. Paridah, M. .; Moradbak, A.; Mohamed, A. .; Owolabi, F. abdulwahab taiwo; Asniza, M.; Abdul Khalid, S. H. . *Applications of RNA-Seq and Omics Strategies - From Microorganisms to Human Health*; 2017; Vol. i;

4. Michele Araújo Pereira, Eddie Luidy Imada, R. L. M. G. Effects of RNA integrity on transcript quantification by total RNA sequencing of clinically collected human placental samples. *FASEB J.* **2017**, *31*, 3298–3308, doi:10.1096/fj.201601031RR.

5. Anders, S.; Pyl, P. T.; Huber, W. HTSeq—a Python framework to work with high-throughput sequencing data. *Bioinformatics* 2014, *31*, 166–169.
